# Anlotinib suppresses metastasis and multidrug resistance *via* dual blockade of MET/ABCB1 in colorectal carcinoma cells

**DOI:** 10.7150/jca.45618

**Published:** 2021-02-16

**Authors:** Lin-Hai Yan, Di Zhang, Si-Si Mo, Hao Yuan, Xian-Wei Mo, Jin-Min Zhao

**Affiliations:** 1 Department of Gastrointestinal Surgery, Guangxi Medical University Cancer Hospital, Nanning 530021, Guangxi Zhuang Autonomous Region, China.; 2 Guangxi Clinical Research Center for Colorectal Cancer, Nanning 530021, Guangxi Zhuang Autonomous Region, China.; 3 Department of Pharmacology, Guangxi Medical University, Nanning 530021, Guangxi Zhuang Autonomous Region, China.

**Keywords:** anlotinb, colorectal carcinoma, metastasis, MET, ABCB1

## Abstract

Anlotinib, a highly selective multi-targeted tyrosine kinase inhibitor (TKI) has therapeutic effects on non-small-cell lung cancer (NSCLC). In this study, the anti-tumor activity and molecular mechanism of anlotinib in metastatic colorectal cancer (mCRC) was explored. The anti-angiogenesis, anti-metastasis, anti-proliferative, and anti-multidrug resistance efficacy of anlotinib were analyzed by using* in vitro* and *in vivo* models of human CRC cells. The results indicated that anlotinib boosted chemo-sensitivity of CRC cells, and restrained its proliferation. Besides the suppression of the MET signaling pathway, anlotinib also inhibited invasion and migration of CRC cells. Furthermore, anlotinib prevented VEGF-induced angiogenesis, N-cadherin (CDH2)-induced cell migration, and reversed ATP-binding cassette subfamily B member 1 (ABCB1) -mediated CRC multidrug resistance in CRC. The CRC liver metastasis and subcutaneously implanted xenograft model testified that anlotinib could inhibit proliferation and liver metastasis in CRC cells. Such an observation suggested that a combination of anlotinib with anti-cancer drugs could attenuate angiogenesis, metastasis, proliferative, and multidrug resistance, which constitutes a novel treatment strategy for CRC patients with metastasis.

## Introduction

Colorectal cancer (CRC) causes most of the cancer-related deaths worldwide 1. The survival rate of two-thirds of CRC patients during early stages of diagnosis improves following an initial radical resection; however, only 11% of such patients with distant metastasis have a five-year survival rate 2. Typically, 20% of CRC patients are reported to have early signs of distant metastasis, whereas 25% of patients develop distant metastasis at later stages of diagnosis 3, 4. Despite advanced diagnostic methods, the development of multidrug resistance for the treatment of CRC patients with metastatic continues to be challenging and causes over 90% of all cancer-related deaths 5, 6. Therefore, studies targeted at novel or alternative therapies for CRC and multidrug resistance are necessary.

Typically, the development of CRC metastasis is driven by angiogenesis and multidrug resistance (MDR) 7, 8. MDR in CRC cells generates resistance to the cytotoxic effects of numerous antineoplastic drugs that reduce the efficacy of cancer chemotherapy 9. Nevertheless, angiogenesis suppression serves as an effective strategy for treating CRC patients with metastasis 10. Single (bevacizumab and cetuximab) and multiple angiogenesis inhibitors (sorafenib and sunitinib) are used to treat advanced CRC 11, 12. However, antiangiogenic therapy was reported to generate elicitation effect in tumor adaptation and progression of the disease that leads to local invasion and distant metastasis 13-15. In this respect, an agent that could simultaneously suppress vascular endothelial growth factor (VEGF) and other pathways involved in metastasis and multidrug resistance is an appropriate therapy for CRC patients with osteosarcoma. Mesenchymal-epithelial transition factor gene (MET) has a remarkable impact on gastrointestinal cancer 16 as it is expressed in more than 70% of the CRC samples. MET inhibition by pharmaceutical or gene therapies treat CRC-MET expressions and is associated with invasion, tumor budding in colorectal cancer (CRC) and CRC pathogenesis 17.

Additionally, MET also regulates cellular migration and drug resistance that influences cancer progression in primary human tumors, such as esophageal cancer, medulloblastoma, and NSCLC 18. Following the result of phase II and phase III clinical trials, it was confirmed that anlotinib exhibited therapeutic effect on NSCLC, soft tissue sarcoma, renal carcinoma, hepatocarcinoma and gastric cancer 19, 20. Furthermore, the blockage of MET/VEGF and ABCB1/ABCG2 pathways disturbs angiogenesis, tumorigenesis, and cancer progression in CRC. The ABC transporter subfamily B member 1 (ABCB1/MDR1/P-glycoprotein), subfamily C member 1 (ABCC1/MRP1) and subfamily G member 2 (ABCG2/BCRP) exert a high impact on the kinetics and dynamics of pharmacological agents and is responsible for the occurrence of multidrug-resistant (MDR) phenotype. They regulate the passage of several antineoplastic drugs across cell membranes that cover taxanes, anthracyclines, vinca alkaloids, and molecular targeting drugs 21, 22. Although ABCB1 and ABCG2 are associated with chemotherapy and malignancy progression 23, 24, they can reduce the intracellular concentration of certain anticancer drugs and pose to be a significant impediment to chemotherapy; hence, a reduction of the expression of the ABC proteins or inhibition of the ABC efflux function by specific inhibitors is critical to reverse MDR in cancerous cells. In this regard, anlotinib, a multi-targeted TKI is reported to prevent phosphorylation-EGFR that suppresses platelet-derived growth factor receptors, including PDGFR, c-Kit, and DDR1. Furthermore, the therapeutic effects of anlotinib require a detailed investigation *via* a comprehensive analysis of cellular function of an animal model, and its molecular mechanism as evident in CRC patients with metastasis.

## Materials and Methods

### Reagents and cell culture

The human colorectal cancer cells HT-29, LoVo, HCT116 and HCT116/L were obtained from the Chinese Academy of Sciences Cell Bank in Shanghai of China. All cells were cultured in DMEM/1640 medium (Invitrogen, Gaithersburg, USA), with 10% FCS (ShiJiQin Biotechnology, Hangzhou, China), as suggested by the suppliers. Anlotinib was obtained from CTTQ-PHARMA (Jiangsu, China).

### Measurement of cell proliferation

For the CCK-8 assay, CRC cells were suspended in 96-well plates at 1 × 10^6^ cells/well, 20 μL of the thymidine analog5-ethynyl-29-deoxyuridine (EdU) solution (Becton-Dickinson, USA) was co-incubated with cells, the results were recorded by using the fluorescence microscope system (Carl Zeiss, Germany). The morphological change of CRC cells treated with anlotinib was observed with NIS microscope imaging software (Nikon, Japan). EdU-positive staining cells be counted in five diverse areas of each well, and we implemented three independent experiments in triplicate.

### Wound-healing migration assay

CRC cells were seeded in 6-well plates at 5 × 10^5^ cells/well. Cells reached full well. The wound bands were subsequently made by using a 10 µl pipette tip (Eppendorf, Germany). Fresh 1640 or DEMN medium (10-15% fetal calf serum) containing anlotinib or PBS was added to the wells. The cells were fixed with 4% paraformaldehyde after 24-72 h, and image of each groups were obtained by employing a fluorescence microscope (Olympus, Japan). The migrated distances of the cells were measured by ImageJ software Version 1.8 (National Institutes of Health, USA), and we implemented three independent experiments in triplicate.

### Transwell assay

Invasion assays were carried out by employing a modified Boyden chamber coated with Matrigel (Transwell Kit). CRC cells were seeded in the upper compartments at a density of 5 × 10^5^ cells/well and treated with 4 μM anlotinib or PBS. Medium (1640/DEMN) containing 2% fetal calf serum (FBS) was added to the upper-chamber, and medium containing 10% FBS was added to the lower-chamber, after which the plates were incubated for 24h - 48h at 37 °C. The CRC cells, which are invaded cells, on the bottom surface of the insert, were fixed with 4% paraformaldehyde and stained with 0.1% crystal violet. Images were obtained by using an inverted microscope (Olympus, Japan), and the invaded CRC cells were counted and we implemented three independent experiments in triplicate.

### Apoptosis analysis by flow cytometry

CRC cells were seeded in 6-well plates at a density of 1×10^6^ cells/well. After incubation for 12h at 37 °C, CRC cells were exposed to indicate concentrations of compounds and incubated at 37°C for another 48 h. And then the cells were trypsinized and washed once with cold PBS. Aliquots of the cells were re-suspended in 500 µL of binding buffer and stained with 5 µL of 7-AAD and 5 µL of PE working solution (Becton-Dickinson, USA) for 15 min at room temperature in the dark. And then analysis was carried out using a Becton-Dickinson FACS Calibur flow cytometer (Becton-Dickinson, USA).

### Immunohistochemical staining (IHC)

All slides were incubated with biotin-label goat anti-mouse IgG (Sigma-Aldrich, St. Louis, USA), followed by horseradish peroxidase (HRP) to label streptavidin. The intensity of immunohistochemical staining was classified into 4 grades (0-III): 0 (negative), I (positive +), II (positive ++), and III (positive +++). The staining extensity was also assessed according to the percentage of positive cancer cells: 0 (1-25%), I (26-50%), II (51-75%), and III (76-100%). The final composite score was the mean of the sum of the intensity and extensity scores. The composite score was regarded as low if the final score was 1-5 and as high if the final composite score was 6-12.

### Quantitative reverse-transcriptase real-time polymerase chain reaction (qRT-PCR)

Total RNA was extracted from each group cells employing TRIzol Reagent Kit (Invitrogen, USA), and then implemented by Quantitative reverse-transcriptase real-time polymerase chain reaction (qRT-PCR). cDNAs were reverse transcription from 2 μg total RNA. S-Table S1 showed the primer sequences used for experiment. The products of qRT-PCR were fastened to agarose gel electrophoresis (Fermentas, USA), and the mRNA expressions were normalized by glyceraldehyde 3-phosphate dehydrogenase (GAPDH) or human gene and protein symbol (β-actin). All mRNA expression was computed by using the 2-^ΔΔ^Ct method, and repeated all experiment three times. The mean value was used as a result of the experiment.

### Western blotting

Cells were collected and lysed by a standard buffer (Biyuntian, China), and total proteins were separated to polyvinylidene difluoride membranes (Biyuntian, China). The membranes were then immunoblotted with the appropriate primary antibody at 4 °C for 12 h, and then co-incubated with HRP conjugated anti-goat second antibody (Santa Cruz, USA). Signals were recorded by using the ECL Western Blotting Kit (Pierce, USA), and quantified the result by Image-Quant LAS 4000 (GE Healthcare, USA). The membranes were co-incubated with first anti-GAPDH and anti-goat second antibody (Santa Cruz, USA) for normalization.

### Xenograft Study

The CRC liver metastasis and subcutaneously implanted xenograft model was reviewed and granted by the Guangxi Medical University Ethic Review Board of Laboratory Animal Care and was implemented in consistence with Guangxi Medical University Animal Facility Guidelines. Aged 6 weeks female BALB/c mice were from the Guangxi Medical University Laboratory Animal Center. For Liver metastasis model, HCT116/L cells stably expressing green luciferase (-GFP) were injected into each mouse's caudal vein. 10 days after injection, when the tumor size reached 100 to 150 mm^3^, the mice were randomly divided into 2 groups (n=4). The mice were treated with anlotinib at a dose of 6 mg/kg once two days by feeder. All the mice were evaluated in two days to testify whether they had developed tumors, and the livers of mice bearing CRC tumor xenografts stably expressing luciferase were analyzed by using an IVIS Lumina Imaging System (Xenogen, USA). For subcutaneously implanted model, The HCT116/L cells (1×10^7)^ were then subcutaneously implanted in the alar of BALB/c mice, when the tumor size reached 500 to 550 mm^3^(TV_0_), the mice were randomly divided into 2 groups (n=5) . The tumor volume was measured and the growth curve was drawn on the basis of the formula (TV = W2 × L/2, L = tumor length and W = tumor width). All the mice were sacrificed at 40 d after CRC tumors were treated with anlotinib, and the implantation of tumors and the livers were finely excised for further study.

### Immunofluorescence assay (IF assay)

CRC cells were seeded on cover slips for IF staining and then stained with primary anti-N-cadherin antibody, primary anti-EGFR antibody, primary anti-Met antibody and primary anti-ABCB1 antibody overnight at 4 °C, respectively. Goat antirabbit IgG were used as secondary antibody. Thereafter, CRC cells were incubated with secondary antibody and the nucleus of CRC cells were stained with DAPI (Sigma, USA). We obtain images via fluorescence microscope (Nikon C2, Japan).

### TUNEL analysis

TUNEL assay was carried out on paraffin sections to evaluate the CRC cell apoptosis. According to manufacturer, *in situ* cell death detection kit (Roche) was used. Under microscope (Leica), TUNEL-positive cells were counted, and apoptotic index (AI) = number of positive cells / total number of cells ×100.

### Statistical analysis

SPSS version 13.0 (SPSS, Chicago, USA) was employed for all statistical analyses. Data are expressed as the mean ± standard error of mean (S.E.M), and statistical significance was implemented using t-tests. The Pearson χ^2^ test was used to compare the frequency of gene overexpression between two paired patients. The Pearson's, Spearman's, and linear regression analyses were used to analyze the relationship between gene levels and variables. A *p* value of < 0.05 was considered significant and was calculated using Origin 8.0 software programs (OriginLab, USA). All analyses were conducted with GraphPad Prism 7 software (GraphPad Software, Inc., San Diego, CA).

## Results

### Anlotinib inhibits CRC cell proliferation and boosts chemosensitivity

**Figure 1:**
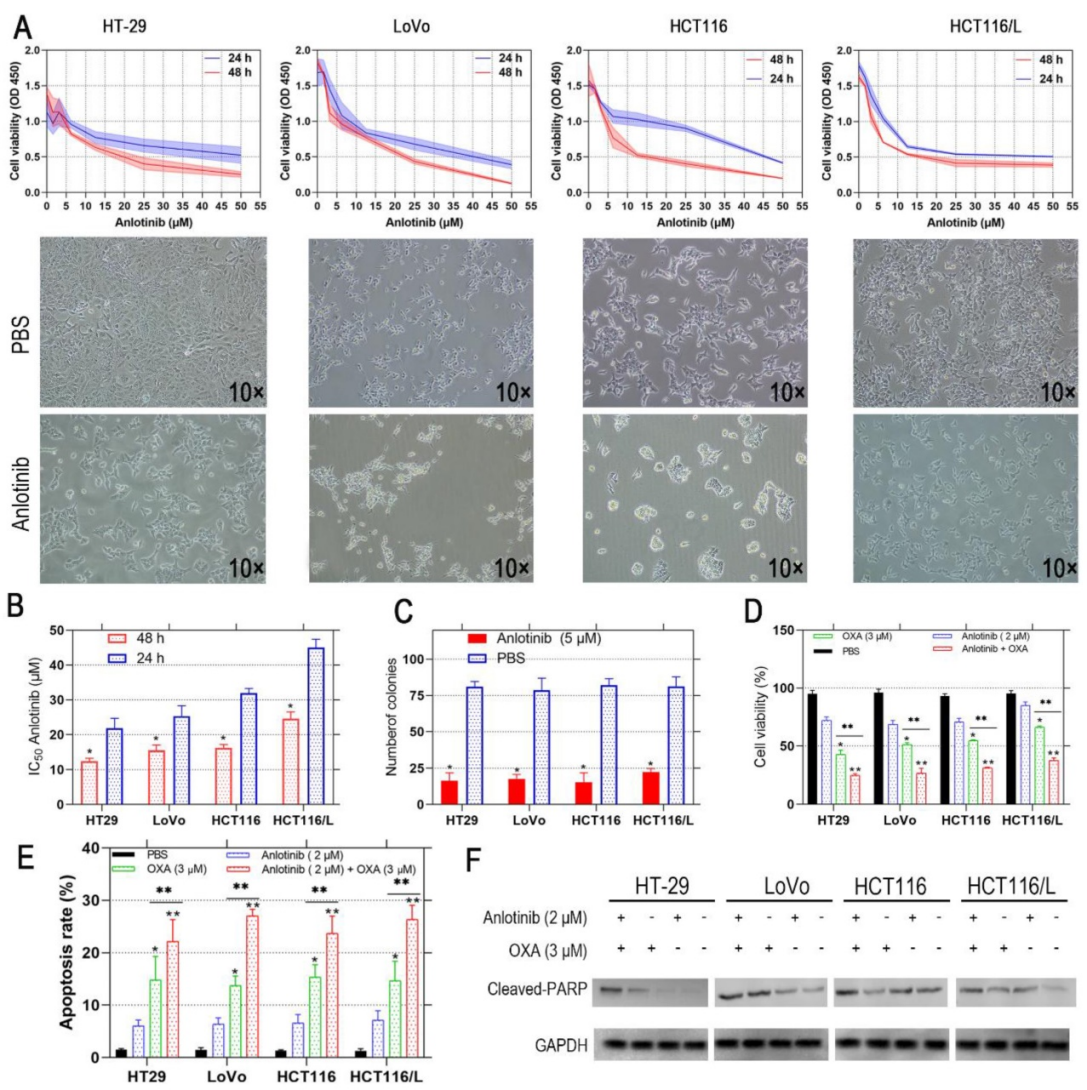
** Anlotinib inhibits cell proliferation and induces cell apoptosis in human CRC cells.** (**A and B**) HT-29, LoVo, HCT116 and HCT116/L cells were treated with anlotinib for 24 or 48 hours. Cell viability was measured by CCK8 kit. (**C**) anlotinib reduces colony formation in CRC cells by clonogenic assay. (**D**) Anlotinib enhances the chemosensitivity of CRC cells to oxaliplatin (OXA). (**E**) The percentages of apoptotic cells in the indicated cell populations are shown in the histogram. (**F**) Western blot assay was used to detect the expression of the apoptosis-related proteins cleaved PARP in HT-29, LoVo, HCT116 and HCT116/L cells, *P<0.05, **P<0.01.

Anlotinib exhibits a therapeutic effect in osteosarcoma and NSCLC 25, 26; however, its effect on CRC has not been investigated. Therefore, we incubated the human CRC cells, i.e., HT-29, LoVo, HCT116, and HCT116/L with anlotinib at the indicated concentrations (5-50 μM) for 24 and 48 hours, respectively. The viability of the CRC cells was assessed by CCK-8 assay, and its effect on CRC cells was evaluated. The results showed that anlotinib inhibited CRC cell proliferation in a time- and dose-dependent manner (Fig. 1A). Anlotinnib inhibited the proliferation of HCT116, and HCT116/L cells with higher IC_50_ values than HT-29 and LoVo cells, respectively (Fig. 1B). The colony formation assays were performed with and without anlotinib. As shown in Fig. 1C, a 50% reduction in the number of colonies were prominent in anlotinib-treated group (5μM) as compared to PBS-treated group, and the incidence of multidrug resistance (MDR) was high in CRC patients with metastasis 27. The CCK-8 assays were used to determine the effect of anlotinib on CRC cells to form oxaliplatin (OXA), which is a first-line anticarcinogen for the treatment of CRC. As shown in Fig. 1D, anlotinib at 2 μM boosted the inhibitory effects of OXA on CRC cell proliferation when OXA was administered at 3 μM. We analyzed the apoptosis of the CRC cells by flow cytometry (FCM) with PI/APC staining to understand the inhibitory effects of anlotinib on CRC cell apoptosis after its treatment with anlotinib at 2 μM and OXA at 3 Μm, respectively. The results suggested that anlotinib could boost the apoptosis rate of the CRC cells (Fig. 1E), whereas western blotting (WB) method indicated that anlotinib decreased OXA-induced PARP cleavage (Fig. 1F). In summary, anlotinib suppressed proliferation and promoted OXA-induced apoptosis in the CRC cells.

### Anlotinib resists angiogenesis and metastasis by inhibition of MET/EGFR pathway

**Figure 2:**
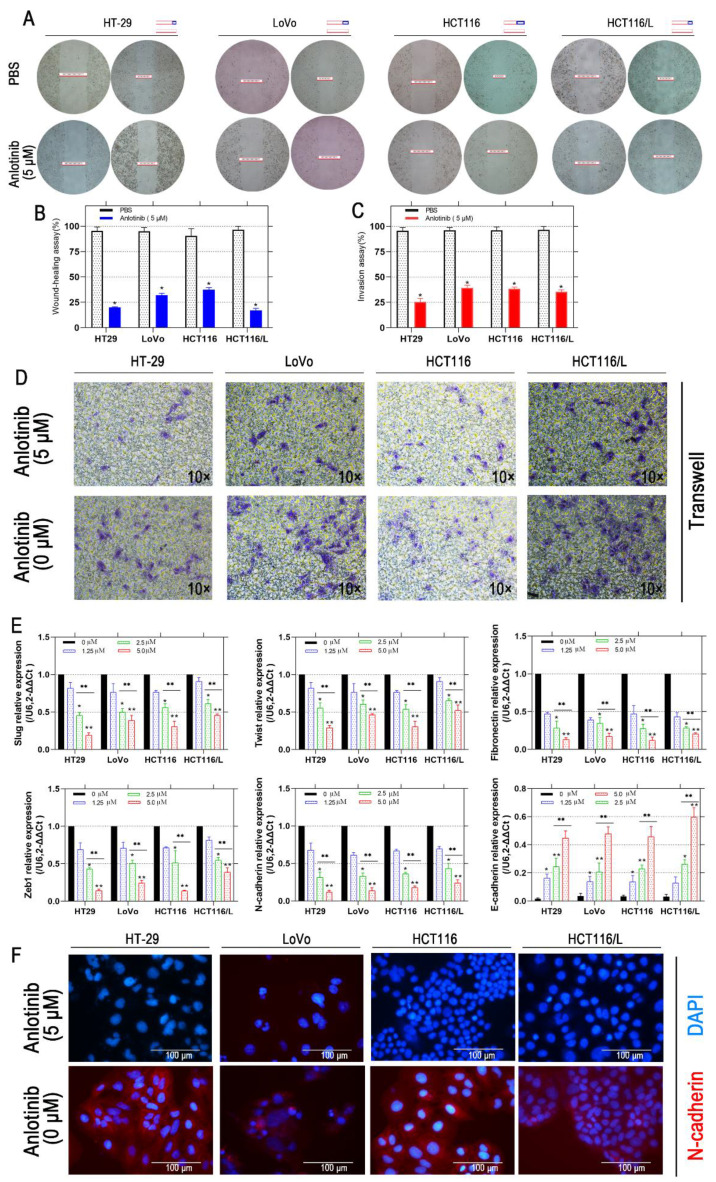
** Anlotinib inhibits migration in CRC cells.** (**A and B**) Cell migration was measured by wound healing assay. The degrees to which the wounds healed in each group are shown in the histogram. (**C and D**) Cell invasion was measured by transwell assay. (**E**) The mRNA levels of metastatic markers were detected using qRT-PCR assay after 24 hours of anlotinib treatment at concentration of 0, 1.25, 2.5 and 5.0 µM. (**F**) N-cadherin levels were detected by IF staining after 24 hours of anlotinib treatment at 5.0 µM. N-cadherin stained red, and the nuclei, which were stained with DAPI, stained blue. *P<0.05, **P<0.01.

**Figure 3:**
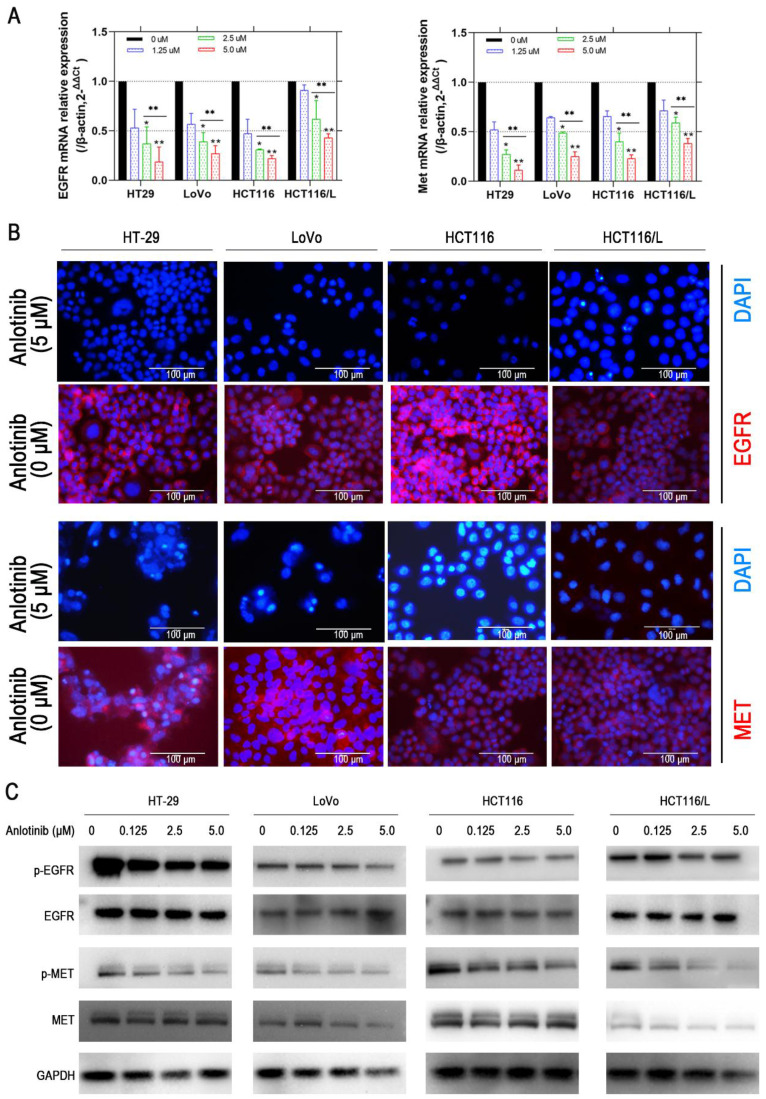
** Anlotinib inhibits MET and EGFR signaling pathway *in vitro*.** (**A**) MET and EGFR mRNA expressions were measured by qRT-PCR. (**B**) MET and EGFR protein levels were detected by IF staining after 24 hours of anlotinib treatment at 5.0 µM in HT-29, LoVo, HCT116 and HCT116/L cells. MET and EGFR protein stained red, and the nuclei, which were stained with DAPI, stained blue. (**C**) Western blot analysis was performed to detect EGFR and MET phosphorylation in CRC cells at concentration of 0, 1.25, 2.5 and 5.0 µM. *P<0.05, **P<0.01.

Liver metastasis was reported to decrease the survival chances of patients afflicted with colorectal cancer 28. Thus, the wound-healing and transwell assays were employed to explore the inhibitory effects of anlotinib on the metastasis of CRC cells. Anlotinib inhibited CRC cells migration and invasion (Fig. 2A-2D). To further investigate the anti-metastasis mechanism of anlotinib, the angiogenesis and metastasis-related gene mRNA levels in the cells were treated with different anlotinib concentrations, i.e. E-cadherin, N-cadherin, ZEB1, Slug, Twist, and Fibronectin, were detected by quantitative reverse transcription-polymerase chain reaction (qRT-PCR). The results demonstrated that anlotinib decreased the expression of Slug, Twist, Fibronectin, ZEB1, and N-cadherin genes, and increased the expression of E-cadherin in dose-dependent manner (1.25, 2.5, and 5.0 μM) (Fig. 2E). Anlotinib decreased the expression of E-cadherin in protein level at 5.0 μM, as indicated by the expression level of E-cadherin (Fig. 2F), a migration key gene. Anlotinib is reported to inhibit MET and EGFR expression in osteosarcoma 26. Consequently, qRT-PCR and western blotting were used to confirm the effect of anlotinib in CRC and explore its mechanism as a downstream effector. IF assays were used to study MET and EGFR protein expressions in HT-29, LoVo, HCT116, and HCT116/L cells treated with PBS or 5 μM anlotinib for 24 h. Anlotinib suppressed EGFR and MET mRNA in anlotinib-treated CRC cells (Fig. 3A) that suppressed MET and EGFR in CRC (or MDR CRC cell) cell membrane (Fig. 3B). Next, the effect of anlotinib treatment on EGFR and MET phosphorylation (Fig. 3C) was also investigated. Following treatment with anlotinib at 1.25, 2.5, or 5.0 μmol/L, the phosphorylation levels of MET and EGFR were suppressed in CRC cells. In summary, anlotinib inhibited the MET and EGFR phosphorylation, and the downstream angiogenesis-, proliferative- and metastasis-related pathway in CRC.

### Anlotinib reverse CRC MDR by blocking the ABCB1/AKT pathway

**Figure 4:**
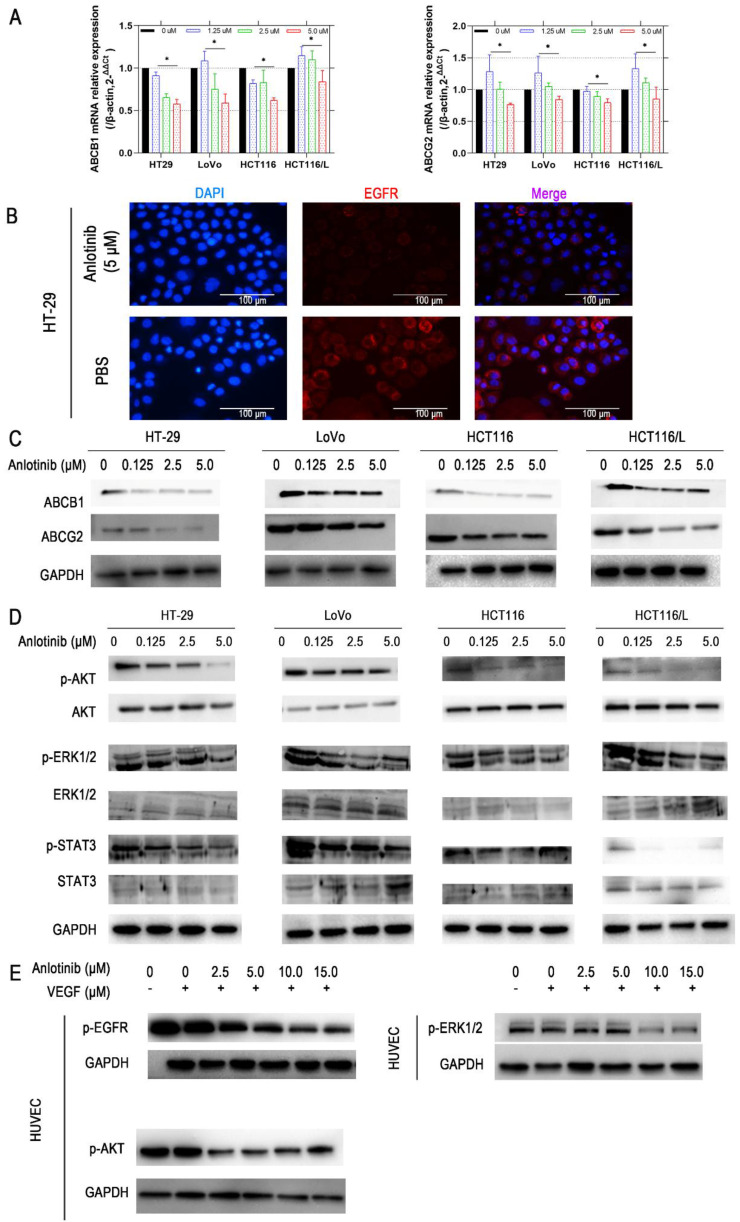
** Anlotinib inhibits ABCB1-induced CRC cell multidrug resistance.** (**A**) ABCB1 and ABCG2 mRNA expressions were measured by qRT-PCR. (**B**) EGFR protein levels were detected by IF staining after 24 hours of anlotinib treatment at 5.0 µM in HT-29, LoVo, HCT116, and HCT116/L cells. (**C**) Western blot analysis was performed to detect ABCB1 and ABCG2 in CRC cells at concentration of 0, 1.25, 2.5 and 5.0 µM. (**D**) Western blot analysis was performed to detect ABCB1-dependent signaling pathway phosphorylation in CRC cells. (**E**) HUVECs were preincubated with 50 ng/ml VEGF for 30 minutes, after which they were incubated with or without anlotinib at concentration of 0, 1.25, 2.5, 5.0, 10.0, and 15.0 µM for 12 hours, Western blot analysis then was performed to detect ABCB1-dependent signaling pathway phosphorylation in CRC cells.

The reversal of ABCB1- and ABCG2-mediated MDR is possible by lowering their expression 29. Consequently, the effects of anlotinib on the protein and mRNA expression level of ABCB1 and ABCG2 in CRC cells were explored. Anlotinib at 1.25, 2.5, or 5.0 μmol/L suppressed the mRNA expression of ABCB1 or ABCG2 in HT-29, LoVo, HCT116, or HCT116/L CRC cells (Fig. 4A). Western blot results were consistent with protein levels in HT-29, LoVo, HCT116, and HCT116/L cells (Fig. 4C). Based on the IF assay, we found out that anlotinib suppressed EGFR in CRC cell (HT-29) membrane (Fig. 4B). Some studies reported that the inhibition of the AKT and ERK1/2 pathways could reverse drug-resistance 30, 31. Hence, we investigated the effect of anlotinib on the levels of total and phosphorylated forms of AKT, ERK1/2, and STAT3 in CRC cells (Fig. 4D). The incubation of cells with anlotinib (0.125-5.0 μmol/L) decreased the expression of phosphorylated forms of AKT, ERK1/2, and STAT3 in all CRC cells, which indicated that the MDR reversal effect of anlotinib in HT-29, LoVo, HCT116, or HCT116/L cells was dependent on the inhibition of AKT, ERK1/2, and STAT3 phosphorylation. Moreover, the cytokine-stimulated tyrosine kinase activity assay confirmed VEGF induced tyrosine autophosphorylation of EGFR, AKT, and ERK1/2 in Human Umbilical Vein Endothelial Cells (HUVECs); however, such changes could be reversed in a concentration-dependent manner by the simultaneous administration of anlotinib at concentration of 2.5, 5.0, 10.0, and 15.0 (Fig. 4E).

### Anlotinib suppresses tumor proliferation and metastasis in CRC liver metastasis xenograft model

**Figure 5:**
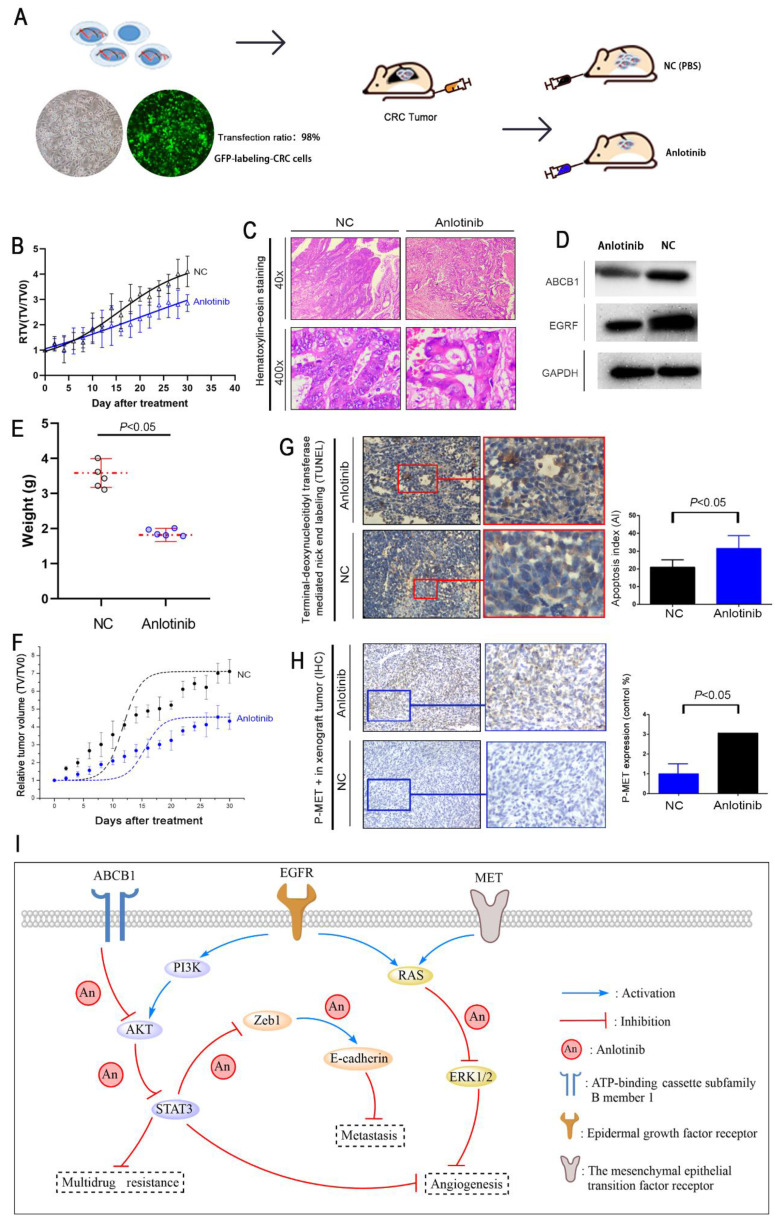
** Anlotinib inhibits tumor growth, metastasis, and angiogenesis in the CRC liver metastasis and subcutaneously implanted xenograft model.** (A) The liver metastasis xenograft model of CRC were established, HCT116/L cells stably expressing green luciferase (-GFP) were injected into the tail veins of all mice, which were treated with an oral dose of 6 mg/kg anlotinib. (**B**) Liver metastasis volumes of anlotinib group was reduced compared with the NC group. (**C**) Hematoxylin-eosin staining of liver tumor metastasis specimens from the NC and anlotinib groups. (**D**) Western blot analysis was performed to detect ABCB1 and EGFR in liver metastasis tumor specimens. The subcutaneously implanted tumor model of HCT116/L cells were also established. (**E and F**) Tumor weights and volumes of anlotinib group were reduced compared with the NC group. (**G**) The percentage of apoptosis was detected by TUNEL assay, the apoptosis was decreased in the anlotinib group compared with the NC group. (**H**) Representative pictures of p-MET immunostaining are shown. (**I**) Molecular pathways involved in anlotinib inhibiting CRC liver metastases. Most notable signaling pathways are EGFR, MET, and ABCB1.

A liver metastasis xenograft model of colorectal cancer was established to evaluate the anti-metastasis effects of anlotinib *in vivo*. Human CRC cell HCT116/L consisting of luciferase (green fluorescence) was injected into the tail veins of all BALB/c mice. For treatment mode, a group of mice received an oral dose of anlotinib, i.e., 6 mg/kg (anlotinib) compared to the PBS-treated group (NC). The growth and location of the metastasis CRC tumor in the livers were monitored, and luciferase imaging was performed every two days (Fig. 5A). The results indicated that anlotinib could inhibit CRC liver metastatic growth in the anlotinib group as compared to the NC group (Fig. 5B). We further examined the liver metastasis nodules in each group by H&E staining, and the results showed that the liver metastasis nodules critically decreased in the anlotinib group as compared to the NC group (Fig. 5C). Western blotting analysis of tumor tissues afflicted with liver metastasis indicated that anlotinib could significantly inhibit EGFR and ABCB1 expression *in vivo* (Fig. 5D). These results were consistent with *in vitro* results. Furthermore, anlotinib also inhibited CRC subcutaneously implanted tumor growth in the anlotinib group as compared to NC group (Fig. 5E-F). We discovered that the percentage of apoptosis in CRC cells decreased in the anlotinib group as compared to the NC group (Fig. 5G). Immunohistochemistry (IHC) analyses of subcutaneously implanted tumor tissues showed that anlotinib could inhibit phospho-MET expression *in vivo* (Fig. 5H). Collectively, these data indicated that anlotinib could suppress tumor metastasis, angiogenesis, and multidrug resistance which reduced MET, EGFR, and ABCB1 expression in CRC liver metastasis and subcutaneously implanted xenograft model (Fig. 5I).

## Discussion

Although the survival rate of stage-I CRC is up to about 90%, the survival rate of metastatic colorectal cancer (mCRC) is low 32. Among all metastatic organs, liver and lungs are two of the most popular site for mCRC. Indeed, the median survival time of mCRC patients after hepatic metastases with the best single/multi-targeted drugs is limited to 6 months 33. Therefore, novel targeted drugs directed against molecules involved in the progression of metastasis and proliferation of CRC cells have emerged as promising therapies. Specifically, EGFR, the plasma membrane molecules VEGF, and BRAF molecules are well-established drug targets, and their inhibitors have shown clinical activity in mCRC 34. Other receptor inhibitors that could increase MDR in mCRC patients remains to be investigated. N-cadherin is associated with the formation of MDR 35, which significantly decreases the expression of mesenchymal markers such as, ZEB1, Fibronectin, Slug, Twist, and increases E-cadherin. The activation of aberrant epithelial-mesenchymal transition (EMT) increases the motility and invasiveness ability of cancerous cells 36, which is characterized by the loss of epithelial cell junction proteins, e.g., E-cadherin, and promotes the expression of mesenchymal markers, e.g., N-cadherin 37. Some recent studies have also reported that the tyrosine kinase receptor MET, encoded by the proto-oncogene c-met, is related to CRC development, and is a poor prognostic factor for CRC patients 17, 38. In fact, the expression of ATP binding cassette subfamily B member-1 (ABCB1) signals an early event in colorectal carcinogenesis and is associated with MDR in mCRC patients 39, 40.

A novel multi-targeted RTKi, anlotinib has shown anti-tumor effects against NSCLC and soft tissue sarcoma 41; meanwhile, its effect on mCRC has not been investigated. In this study, we demonstrated that anlotinib could exert anti-angiogenesis, anti-metastasis, anti-proliferative and anti-multidrug resistance effects by blocking EGFR, MET, and ABCB1 *in vivo* and *in vitro*. The MET signaling pathway affected CRC tumor growth 42, and anlotinib inhibited the growth of CRC cells by regulating MET-induced apoptosis in both dose- and time-dependent manner. Moreover, EGFR-TKI (e.g. apatinib and rapamycin) enhanced the chemosensitivity of cancer 43, 44, which suggested that anlotinib could increase the sensitivity of CRC cells to OXA. Typically, anlotinib suppresses CRC cellular proliferation that increases its chemosensitivity. Our results indicated that anlotinib could also suppress clinical metastasis and MDR in mCRC. Furthermore, the imbalance between pro-angiogenic and anti-angiogenic factors as regulated by Vascular endothelial growth factors (VEGFs) and their receptors (VEGFRs) could also lead to tumorous growth and metastatic 45, 46. The phosphorylation of VEGFR2 and the activation of the downstream signaling molecules (e.g. STAT3, Akt, and Erk-1/2) caused by binding of VEGF to VEGFR2 were reported to promote cancerous cell growth and migration 47. Therefore, the inhibition of EGFR/Akt /STAT3 pathway could be a strategy for treating mCRC 48. In our study, we assessed EGFR and MET inhibitory effects of anlotinib in mCRC, and identified that anlotinib could suppress VEGF-induced activation of STAT3, Akt, and Erk-1/2. The inhibition of VEGF leads to hypoxia-induced MET receptor activation, which caused tumor revascularization and invasive enhancement. These findings indicated that inhibiting MET and VEGF pathway could improve anti-tumor effect as compared to single target therapy 49.

The ABC transporters affect multidrug resistance (MDR *via* the release of doxorubicin and paclitaxel, which are substrates of the ABCB1 transporter; whereas, ABCG2 transporter expels dasatinib, imatinib, and nilotinib 23, 50, 51. Interesting, several TKIs were reported to interact with ABC transporters. For example, imatinib acts as an ABCB1 substrate, while nilotinib could interact with ABCC1; furthermore, imatinib and nilotinib are confirmed to be substrates and inhibitors of ABCG2 and reversed ABCB1-mediated drug efflux, respectively 52. TKIs (e.g. gefitinib 53, 54, erlotinib 55, and vandetanib 56) were also reported to suppress ABCB1 and ABCG2 expression in cancerous cells. Consequently, the cytotoxic potential of ABCB1 and ABCG2 substrates and accumulated OXA in ABCB1- or ABCG2-expressing CRC cells was established in our study. Our results indicated that anlotinib enhanced the intracellular accumulation of OXA in MDR cells (HCT116/L) and exhibited a strong effect on anti-angiogenesis, anti-metastasis, anti-proliferative and anti-multidrug resistance in CRC by blocking the EGFR, MET, and ABCB1 signaling pathways, which exhibited a robust pharmacological effect in the CRC Liver metastases xenograft model. Anlotinib also demonstrated a broad-spectrum antitumor potential that allowed its co-administration with immune targeted drugs or conventional anticancer drugs that improved survival outcomes of mCRC patient.

## Conclusions

Our findings identify that anlotinib exhibited a strong effect on anti-angiogenesis, anti-metastasis, anti-proliferative and anti-multidrug resistance in CRC by blocking the EGFR, MET, and ABCB1 signaling pathways, which exhibited a robust pharmacological effect in the CRC liver metastases xenograft model. Anlotinib also demonstrated a broad-spectrum antitumor potential that allowed its co-administration with immune targeted drugs or conventional anticancer drugs that improved survival outcomes of mCRC patient.

## Supplementary Material

Supplementary table S1.
