# Supplementary material for: Anlotinib suppresses metastasis and multidrug resistance via dual blockade of MET/ABCB1 in colorectal carcinoma cells
Source: J Cancer. 2021 Feb 16;12(7):2092–104. doi: 10.7150/jca.45618 (PMC7974540; doi:10.7150/jca.45618)
Supplement: Supplementary file 1 — Supplementary table S1. [file jcav12p2092s1.pdf]

Supplementary Table 1. The base sequence of primers for RT-PCR

Gene	Primer	Base sequence (5'- > -3')
GAPDH (1)	Forward	GATTCCACCCATGGCAAATTCC
	Reverse	GCATCGCCCCACTTGATTTT
GAPDH (2)	Forward	ATGGCAAATTCCATGGCACCG
	Reverse	CAGCATCGCCCCACTTGATT
β-actin	Forward	GGAAGAAGAGATCGCCGC
	Reverse	ATGGAAGGAAACACGGCTCG
EGFR	Forward	AAAACCGGACTGAAGGAGCTG
	Reverse	GATGCTCTCCACGTTGCACA
FGFR	Forward	CACATCCAGTGGCTCAAGCA
	Reverse	CTAGCTCCTTGTCGGTGGTG
PDGFR-a	Forward	TGTGGGACATTCATTGCGGA
	Reverse	AAGCTGGCAGAGGATTAGGC
PDGFR-b	Forward	CAGCTCTGGCCCTCAAAGG
	Reverse	GAACGAAGGTGCTGGAGACA
ABCB1 (1)	Forward	GGAGGCCAACATACATGCCT
	Reverse	AGGCTGTCTAACAAGGGCAC
ABCB1 (2)	Forward	ACAGAGGGGATGGTCAGTGT
	Reverse	TCACGGCCATAGCGAATGTT
ABCB2 (1)	Forward	AAACCTGGTCTCAACGCCAT
	Reverse	TCGCGGTGCTCCATTTATCA
ABCB2 (2)	Forward	TCCCCAGGCCTCTATAGCTC
	Reverse	AACACTGGTTGGTCGTCAGG
AKT (1)	Forward	TACGAGATGATGTGCGGTCG
	Reverse	CAGCCCTGAAAGCAAGGACT
AKT (2)	Forward	CAGGATGTGGACCAACGTGA
	Reverse	AAGGTGCGTTCGATGACAGT
ERK1 (1)	Forward	ACTCCAAAGCCCTTGACCTG

	Reverse	GGGACTGGCCCACCTCAT
ERK1 (2)	Forward	AGACTCCAAAGCCCTTGACC
	Reverse	GGACTGGCCCACCTCATC
ERK2 (1)	Forward	GGCTGTTCCCAAATGCTGAC
	Reverse	CTCGTCACTCGGGTCGTAAT
ERK2 (2)	Forward	CGTGTTGCAGATCCAGACCA
	Reverse	GCCAGAATGCAGCCTACAGA
slug	Forward	CGGGGGAGAAGCCTTTTTCT
	Reverse	CACAGCAGCCAGATTCCTCA
TWIST	Forward	GGAAGATCATCCCCACGCTG
	Reverse	GCTCTGGAGGACCTGGTAGA
p-MET	Forward	CTACCACGCCTTCTCAGCAA
	Reverse	GGTCCGAGAGGCATTCACAA
MET	Forward	TCCTCTGGGAGCTGATGACA
	Reverse	CTGGGCAGTATTCGGGTTGT
Caspase-3	Forward	CCTGGTTCATCCAGTCGCTT
(1)	Reverse	TCTGTTGCCACCTTTCGGTT
Caspase-3	Forward	GCAGCAAACCTCAGGGAAAC
(2)	Reverse	CACCATGGCTCAGAAGCACA
PARP	Forward	CCTCCTCCTACCTCTGGTGC
	Reverse	CTGGACAGATGCCTTTCGCT
N-cadherin	Forward	GGGAATCCGACGAATGGATGA
	Reverse	GAGCCACTGCCTTCATAGTCA
Zeb1 (1)	Forward	TGAGGGTGCACAAGAAGAGC
	Reverse	GCGCAAGACAAGTTCAAGGG
Zeb1 (2)	Forward	CACTGCCCAGTTACCCACAA
	Reverse	CAGGGCTGACCGTAGTTGAG
E-cadherin	Forward	TCCATTTCTTGGTCTACGCCT
(1)	Reverse	GGAGTTGGGAAATGTGAGCAA

E-cadherin	Forward	CCACCAAAGTCACGCTGAATAC
(2)	Reverse	TGGGAGGAATAACCCAGTCTCT
Vimentin	Forward	TGGCACGTCTTGACCTTGAA
(1)	Reverse	GGCTTGGAAACATCCACATCG
Vimentin	Forward	CACGTCTTGACCTTGAACGC
(2)	Reverse	TCAGGCTTGGAAACATCCACA
Fibronectin	Forward	TAACCAACTTCCTGGTGCGT
(1)	Reverse	AGAGGTGTGCTCTCATGTTGT
Fibronectin	Forward	CTCCCACTGACCTGCGATTC
(2)	Reverse	ACGCACCAGGAAGTTGGTTA
